# Images from Bits: Non-Iterative Image Reconstruction for Quanta Image Sensors

**DOI:** 10.3390/s16111961

**Published:** 2016-11-22

**Authors:** Stanley H. Chan, Omar A. Elgendy, Xiran Wang

**Affiliations:** 1 School of Electrical and Computer Engineering, Purdue University, 465 Northwestern Ave, West Lafayette, IN 47907, USA; oelgendy@purdue.edu (O.A.E.); wang470@purdue.edu (X.W.); 2 Department of Statistics, Purdue University, 250 N. University Street, West Lafayette, IN 47907, USA

**Keywords:** single-photon image sensor, quanta image sensor (QIS), image reconstruction, quantized Poisson statistics, image denoising, Anscombe Transform, maximum likelihood estimation (MLE)

## Abstract

A quanta image sensor (QIS) is a class of single-photon imaging devices that measure light intensity using oversampled binary observations. Because of the stochastic nature of the photon arrivals, data acquired by QIS is a massive stream of random binary bits. The goal of image reconstruction is to recover the underlying image from these bits. In this paper, we present a non-iterative image reconstruction algorithm for QIS. Unlike existing reconstruction methods that formulate the problem from an optimization perspective, the new algorithm directly recovers the images through a pair of nonlinear transformations and an off-the-shelf image denoising algorithm. By skipping the usual optimization procedure, we achieve orders of magnitude improvement in speed and even better image reconstruction quality. We validate the new algorithm on synthetic datasets, as well as real videos collected by one-bit single-photon avalanche diode (SPAD) cameras.

## 1. Introduction

### 1.1. Quanta Image Sensor

Since the birth of charge coupled devices (CCD) in the late 1960s [1] and the complementary metal-oxide-semiconductor (CMOS) active pixel sensors in the early 1990s [2], the pixel pitch of digital image sensors has been continuously shrinking [3]. Shrinking the pixel pitch is intimately linked to the need of increasing image resolution, reducing power consumption and reducing the size and weight of cameras. However, as pixel pitch shrinks, the amount of photon flux detectable by each pixel drops, leading to reduced signal strength. In addition, the maximum number of photoelectrons that can be held in each pixel, known as the full-well capacity, also drops. Small full-well capacity causes reduced maximum signal-to-noise ratio and lowers the dynamic range of an image [4]. Therefore, pushing for smaller pixels, although feasible in the near future, will become a major technological hurdle to new image sensors.

A quanta image sensor (QIS) is a class of solid-state image sensors originally proposed by Eric Fossum as a candidate solution for sub-diffraction-limit pixels. The sensor was first named the digital film sensor [5] and later the quanta image sensor [6,7,8,9] (see [10] for a more comprehensive discussion of the history). A similar idea to QIS was developed a few years later by EPFL (École Polytechnique Fédérale de Lausanne, Lausanne, Switzerland), known as the Gigavision camera [11,12,13]. In the past few years, research groups at the University of Edinburgh (Edinburgh, UK) [14,15,16], as well as EPFL [17,18] have made new progresses in QIS using binary single-photon avalanche diode (SPAD) cameras. In the industry, Rambus Inc. (Sunnyvale, CA, USA) is developing binary image sensors for high dynamic range imaging [19,20,21]. For the purpose of this paper, we shall not differentiate these sensors, but refer to them generally as the QIS, because their underlying mathematical principles are similar.

The working principle of QIS is as follows: In CCD and CMOS, one considers each pixel as a “bucket” that collects and integrates photoelectrons. The bucket is partitioned in QIS into thousands of nanoscale cells referred to as “jots”. Each jot is capable of detecting a single photon to generate a binary response indicating whether the photon count is above or below a certain threshold *q*. If the photon count is above *q*, the sensor outputs a “1”; If the photon count is below *q*, the sensor outputs a “0”. QIS has a very small full-well capacity because it is not designed to accumulate photons. Since the binary response is generated as soon as the number of photons exceeds the threshold, QIS can be operated at very high speed. For example, using single-photon avalanche diodes (SPAD), one can achieve 10k frames per second with a spatial resolution of 320×240 pixels [14] or even 156k frames per second with a spatial resolution of 512×128 pixels [17]. For a higher spatial resolution, Massondian et al. [9] reports a QIS operating at 1000 frames per second for a spatial resolution of 1376×768 pixels.

From a signal processing perspective, the challenge of QIS is the extremely lossy process using binary measurements to acquire the light intensity. In order to compensate for the loss, QIS over-samples the space by using a large number of jots and takes multiple exposures in time. This technique is similar to the classic approach in oversampled analog-to-digital conversions.

### 1.2. Scope and Contribution

The theme of this paper is about how to reconstruct images from the one-bit quantized measurements. Image reconstruction is a critical component of QIS, for without such an algorithm, we will not be able to form images. However, unlike classical Poisson image recovery problems where solutions are abundant [22,23,24,25,26,27], the one-bit quantization of QIS makes the problem uniquely challenging, and there is a limited number of existing methods [28,29,30,31]. Another challenge we have to overcome is the complexity of the algorithm, which has to be low enough that we can put them on cameras to minimize power consumption, memory consumption and runtime. Numerical optimization algorithms are generally not recommended if they are iterative and require intensive computation for every step.

The main contribution of this paper is a non-iterative image reconstruction algorithm for QIS data. We emphasize the non-iterative nature of the algorithm as it makes the algorithm different from existing methods. The new algorithm is based on a transform-denoise framework. The idea is to apply a variance stabilizing transform known as the Anscombe transform [32] to convert a sum of one-bit quantized Poisson random variables to binomial random variables with equal variances. When variance is stabilized, standard image denoising algorithms can be applied to smooth out the noise in the image. Transform-denoise is a single-pass algorithm with no iteration. Empirically, we find that the new algorithm achieves two orders of magnitude improvement in speed and provides an even better reconstruction result than existing iterative algorithms.

The rest of the paper is organized as follows. First, in Section 2, we present the imaging model of QIS. We discuss how the light intensity of the scene is over-sampled and what statistics do the one-bit quantized measurements have. Unlike existing works, which typically assume a quantization level q=1, we make no assumption about *q*, except that it is a positive integer. This requires some discussion about the incomplete Gamma function. We also discuss why a simple summation over a local spatial-temporal volume is insufficient to reconstruct images. Section 3 presents the main algorithm. We discuss the concept of variance stabilizing transform, its derivation and its limitations. We also discuss how various image denoising algorithms can be plugged into the framework. In Section 4, we present experimental results. There are two sets of data we will discuss. One is a synthetic dataset in which we can objectively measure the reconstruction quality. The other one is a set of real videos captured by SPAD cameras. We compare the proposed algorithm with existing methods. Proofs of major theorems are given in the Appendix A.

## 2. QIS Imaging Model

In this section, we provide a quick overview of the QIS imaging model. A similar description of the model was previously discussed in [13,29]. For notational simplicity, we consider one-dimensional signals. The extension to the two-dimensional images is straightforward. Furthermore, we follow [5] by referring to sub-pixels of a QIS as “jots”.

**Figure 1:**
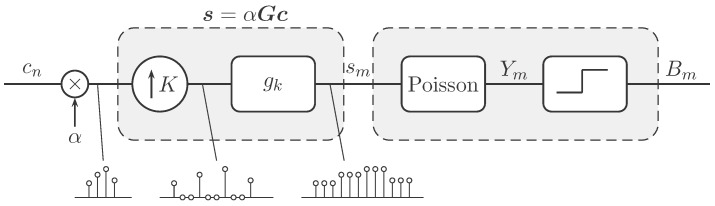
Block diagram of the QIS imaging model. An input signal $c_{n}\in \left[0,1\right]$ is scaled by a constant α>0. The first part of the block diagram is the upsampling (↑K) followed by a linear filter $\left\{g_{k}\right\}$. The overall process can be written as s=αGc. The second part of the block diagram is to generate a binary random variable $B_{m}$ from Poisson random variable $Y_{m}$. The example at the bottom shows the case where K=3.

The mathematics of QIS is built upon two concepts: (1) a spatial oversampling process to model the acquisition by the sensor; and (2) a quantized Poisson process to model the photon arrivals. The block diagram of the model is summarized in Figure 1.

### 2.1. Oversampling Mechanism

We represent the light intensity of a scene using a digital signal $c={\left[c_{0},c_{1},\ldots ,c_{N-1}\right]}^{T}$. To avoid the ambiguity of the scaling, we assume that $c_{n}$ is normalized so that $c_{n}\in \left[0,1\right]$ for n=0,1,…,N−1. A fixed and known constant α>0 is multiplied to scale $c_{n}$ to the proper range.

QIS is a spatial oversampling device. For an *N*-element signal c, QIS uses M≫N number of jots to acquire c. Thus, every $c_{n}$ in the signal c is sampled by M/N jots. The ratio $K\overset{\mathrm{def}}{=}M/N$ is called the spatial oversampling factor. To model the oversampling process, we follow [13] by considering an upsampling operator (↑K) and a low-pass filter with filtering coefficients $\left\{g_{k}\right\}$, as shown in Figure 1. Since upsampling and low-pass filtering are linear operations, the overall process can be compactly expressed using a matrix-vector multiplication

(1) s=αGc,

where $s={\left[s_{0},s_{1},\ldots ,s_{M-1}\right]}^{T}$ is the light intensity arriving at those *M* jots, and the matrix $G\in R^{M\times N}$ encapsulates the upsampling process and the linear filtering.

There are a variety of choices for the low-pass filter depending on which filter provides a better model of the light intensity. In this paper, we make the following assumption about the low-pass filter.

**Assumption** **1.** *We assume that $\left\{g_{k}\right\}$ is a boxcar function, such that $g_{k}=1/K$ for k=0,…,K−1. Consequently, the matrix G is*

(2) $$G=\frac{1}{K}I_{N\times N}⊗1_{K\times 1},$$

*where $1_{K\times 1}$ is a K-by-one all one vector, $I_{N\times N}$ is an N-by-N identity matrix and* ⊗ *denotes the Kronecker product.*

Intuitively, what Assumption 1 does is to assume that the light intensity is piecewise constant. As will be discussed in Section 3, this is important for us to derive simple algorithms.

### 2.2. Quantized Poisson Observation

At the surface of the *m*-th jot, the light intensity $s_{m}$ generates $y_{m}$ photons according to the Poisson distribution

(3) $$P\left\{Y_{m}=y_{m}\;;\;s_{m}\right\}=\frac{s_{m}^{y_{m}}e^{-s_{m}}}{y_{m}!},$$

where $Y_{m}$ denotes the Poisson random variable at the *m*-th jot and $y_{m}$ denotes the realization of $Y_{m}$.

The one-bit quantization of QIS is a truncation of $y_{m}$ with respect to a threshold $q\in Z^{+}$. Precisely, the observed one-bit measurement at the *m*-th jot is

(4) $$b_{m}\overset{\mathrm{def}}{=}\left\{\begin{array}{cc} 1, & \;\mathrm{if}\;y_{m}\geq q,\\ 0, & \;\mathrm{if}\;\mathrm{otherwise}. \end{array}\right.$$

Denoting $B_{m}$ the random variable of the one-bit measurement at the *m*-th jot, it follows that the probability of observing $B_{m}=1$ is

(5) $$P\left\{B_{m}=1\;;\;s_{m}\right\}=P\left\{Y_{m}\geq q\;;\;s_{m}\right\}=\sum_{k=q}^{\infty }\frac{s_{m}^{k}e^{-s_{m}}}{k!},$$

and the probability of observing $B_{m}=0$ is $P\left\{B_{m}=0\;;\;s_{m}\right\}=\sum_{k=0}^{q-1}\frac{s_{m}^{k}e^{-s_{m}}}{k!}$.

For general *q*, keeping track of the sum of exponentials in Equation (5) could be cumbersome. To make our notations simple, we adopt a useful function called the incomplete Gamma function [33], defined as follows.

**Definition** **1.** *The incomplete Gamma function $\Psi_{q}:R^{+}\to \left[0,1\right]$ for a fixed threshold $q\in Z^{+}$ is defined as*

(6) $$\Psi_{q}\left(s\right)\overset{\mathrm{def}}{=}\frac{1}{\Gamma (q)}\int_{s}^{\infty }t^{q-1}e^{-t}dt=\sum_{k=0}^{q-1}\frac{s^{k}e^{-s}}{k!},$$

*where Γ(q)=(q−1)! is the standard Gamma function evaluated at q.*

The incomplete Gamma function is continuous in *s*, but discrete in *q*. For every fixed *s*, $\Psi_{q}\left(s\right)$ is the cumulative distribution of a Poisson random variable evaluated at q−1. When *q* is fixed, $\Psi_{q}$ is the likelihood function of the random variable $B_{m}$. In this paper, we will focus on the latter case where *q* is fixed so that $\Psi_{q}$ is a function of *s*. Since $\Psi_{q}$ is continuous, the derivative of $\Psi_{q}\left(s\right)$ is available:

**Property** **1.** *The first order derivative of $\Psi_{q}\left(s\right)$ is*

(7) $$\frac{d}{ds}\Psi_{q}\left(s\right)=-\frac{e^{-s}s^{q-1}}{\Gamma (q)}$$

With the incomplete Gamma function, we can rewrite Equation (5) as

(8) $$\begin{array}{c} P\left\{B_{m}=1\;;\;s_{m}\right\}=1-\Psi_{q}\left(s_{m}\right),\;\mathrm{and}\;P\left\{B_{m}=0\;;\;s_{m}\right\}=\Psi_{q}\left(s_{m}\right). \end{array}$$

**Example** **1.** *When q=1, the incomplete Gamma function becomes $\Psi_{q}\left(s_{m}\right)=e^{-s_{m}}$. In this case,*

$$\begin{array}{c} P\left\{B_{m}=1\;;\;s_{m}\right\}=1-e^{-s_{m}}\;and\;P\left\{B_{m}=0\;;\;s_{m}\right\}=e^{-s_{m}}. \end{array}$$

### 2.3. Image Reconstruction for QIS

We consider a multiple exposure setting. Assuming that the scene is stationary, the measurement we obtain is a sequence of binary bit maps $\left\{b_{m,t}\;|\;m=0,\ldots ,M-1,\;\mathrm{and}\;t=0,\ldots ,T-1\right\}$, where the first index *m* runs through the *M* jots spatially, and the second index *t* runs through the *T* frames in time. The image reconstruction task is to find the signal $c={\left[c_{0},\ldots ,c_{N-1}\right]}^{T}$ that best explains $\left\{b_{m,t}\right\}$. Translating into a probabilistic framework, this can be formulated as maximizing the likelihood:

(9) $$\begin{array}{cc} \hat{c} & =\underset{c}{\mathrm{argmax}}\;\;\prod_{t=0}^{T-1}\prod_{m=0}^{M-1}P{\left[B_{m,t}=1\;;\;s_{m}\right]}^{b_{m,t}}P{\left[B_{m,t}=0\;;\;s_{m}\right]}^{1-b_{m,t}},\;\;\mathrm{subject}\;\mathrm{to}\;\;s=\alpha Gc,\\ & =\underset{c}{\mathrm{argmax}}\;\;\prod_{t=0}^{T-1}\prod_{m=0}^{M-1}{\left(1-\Psi_{q}\left(s_{m}\right)\right)}^{b_{m,t}}\Psi_{q}{\left(s_{m}\right)}^{1-b_{m,t}},\;\;\mathrm{subject}\;\mathrm{to}\;\;s=\alpha Gc, \end{array}$$

where the constraint s=αGc follows from Equation (1). Taking the logarithm on the right-hand side of Equation (9), the maximization becomes

(10) $$\hat{c}=\underset{c}{\mathrm{argmax}}\;\;\sum_{t=0}^{T-1}\sum_{m=0}^{M-1}\left\{b_{m,t}\log\left(1-\Psi_{q}\left(s_{m}\right)\right)+\left(1-b_{m,t}\right)\log\Psi_{q}\left(s_{m}\right)\right\},\;\;\mathrm{subject}\;\mathrm{to}\;\;s=\alpha Gc.$$

The optimization in Equation (10) is known as the maximum likelihood estimation (MLE). As shown in [13], the objective function of Equation (10) is concave (for any *q*), and therefore, the global maximum exists and is unique. When G satisfies Assumption 1, the closed form solution is available, and we will show it in Section 3. However, for general G or if we include additional constraints to enforce the smoothness of the solution, e.g., using total variation [34] or sparsity [35], then numerical algorithms are required. Some existing methods include the gradient descent method [13], Newton method [28] and the alternating direction method of multipliers [29]. These algorithms are iterative, and the computation for each iteration is costly.

## 3. Non-Iterative Image Reconstruction

In this section, we present a non-iterative image reconstruction algorithm for QIS. There are three components behind the algorithm. The first is the closed-form solution of Equation (10), which is coherent to [13]. The second component is a variance stabilizing transform that transforms the sum of one-bit quantized Poisson random variables to a binomial random variable with equal variances. The transformation is known as the Anscombe transform, named after the English statistician Frank Anscombe (1918–2001). The third idea is the application of an off-the-shelf image denoiser.

### 3.1. Component 1: Approximate MLE

Because of the piecewise constant property of Assumption 1, the sequence $\left\{b_{m,t}\right\}$ can be partitioned into *N* blocks where each block contains K×T binary bits. This leads to a decomposition of Equation (10) as

(11) $$\hat{c}=\underset{c}{\mathrm{argmax}}\;\;\sum_{t=0}^{T-1}\sum_{n=0}^{N-1}\sum_{k=0}^{K-1}\left\{b_{Kn+k,t}\log\left(1-\Psi_{q}\left(\frac{\alpha c_{n}}{K}\right)\right)+\left(1-b_{Kn+k,t}\right)\log\Psi_{q}\left(\frac{\alpha c_{n}}{K}\right)\right\},$$

where the subsequence $B_{n,t}\overset{\mathrm{def}}{=}\left\{b_{Kn,t},\ldots ,b_{Kn+(K-1),t}\right\}$ denotes the *n*-th block of the *t*-th frame. Define

(12) $$S_{n}\overset{\mathrm{def}}{=}\sum_{t=0}^{T-1}\sum_{k=0}^{K-1}b_{Kn+k,t}$$

be the sum of the bits (i.e., the number of one’s) in $B_{n,t}$. Then, Equation (11) becomes

(13) $$\begin{array}{c} \hat{c}=\underset{c}{\mathrm{argmax}}\;\;\sum_{n=0}^{N-1}S_{n}\log\left(1-\Psi_{q}\left(\frac{\alpha c_{n}}{K}\right)\right)+\left(L-S_{n}\right)\log\Psi_{q}\left(\frac{\alpha c_{n}}{K}\right), \end{array}$$

where L=KT. The maximum of Equation (13) is attained when each individual term in the sum attains its maximum. In this case, the closed form solution of Equation (13), for every *n*, can be derived as in Proposition Equation 1.

**Proposition** **1.** *The solution of Equation* (13) *is*

(14) $${\hat{c}}_{n}=\frac{K}{\alpha }\Psi_{q}^{-1}\left(1-\frac{S_{n}}{L}\right),\;\;n=0,\ldots ,N-1,$$

*where L=KT, K is the spatial oversampling factor and T is the number of exposures.*

**Proof.**  See Appendix A. ☐

**Figure 2:**
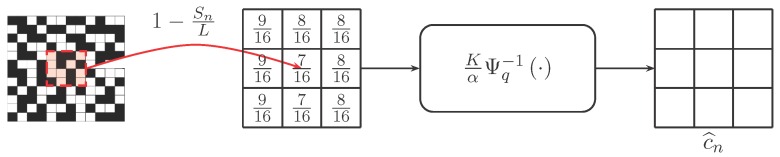
Pictorial interpretation of Proposition 1: Given an array of one-bit measurements (black = 0, white = 1), we compute the number of ones within a block of size *K*. Then, the solution of the MLE problem in Equation (13) is found by applying an inverse incomplete Gamma function $\Psi_{q}^{-1}\left(\cdot \right)$ and a scaling factor K/α.

It would be instructive to illustrate Proposition 1 using a figure. Figure 2 shows the case when T=1, i.e., a single exposure, and K=16. The one-bit measurements are first averaged to compute the number of ones within a block of size *K*. Then, applying the inverse incomplete Gamma function $\Psi_{q}^{-1}\left(\cdot \right)$ and a scaling constant K/α, we obtain the solution ${\hat{c}}_{n}$.

**Figure 3:**
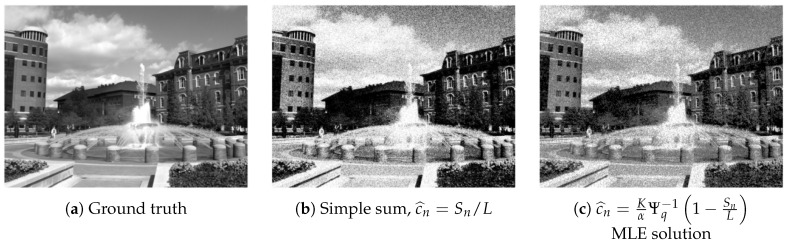
Image reconstruction using synthetic data. In this experiment, we generate one-bit measurements using a ground truth image (**a**) with α=160, q=5, K=16, T=1 (so L=16). The result shown in (**b**) is obtained using the simple summation, whereas the result shown in (**c**) is obtained using the MLE solution. It can be seen that the simple summation has a mismatch in the tone compared to the ground truth.

Proposition 1 shows why a simple summation $S_{n}/L$ is inadequate to achieve the desired result, although such summation has been used in [18,36]. By only summing the number of ones, the resulting value $S_{n}/L$ is the empirical average of these one-bit measurements. Since the probability of drawing a one in QIS follows a quantized Poisson distribution and not a Bernoulli distribution, the nonlinearity due to the quantized Poisson distribution must be taken into account. A comparison of the ground truth image, the summation result and the MLE solution is shown in Figure 3.

As an immediate corollary of Proposition 1, we can simplify the inverse incomplete Gamma function when q=1. This result is sometimes known as the exponential correction function [37].

**Corollary** **1.** *When q=1, the MLE solution of the n-th pixel ${\hat{c}}_{n}$ is*

(15) $${\hat{c}}_{n}=-\frac{K}{\alpha }\log\left(1-\frac{S_{n}}{L}\right),\;\;n=0,\ldots ,N-1,$$

*where $S_{n}$ is the number of ones in the n-th block (See Equation.* (12)*) and L=KT is the total number of pixels in the block.*

**Proof.**  The result follows from the fact that $\Psi_{q}\left(s\right)=e^{-s}$ for q=1. Thus, $\Psi_{q}^{-1}\left(s\right)=-\log s$. ☐

### 3.2. Component 2: Anscombe Transform

The MLE solution $\hat{c}={\left[{\hat{c}}_{0},\ldots ,{\hat{c}}_{N-1}\right]}^{T}$ computed through Proposition 1 is noisy, as illustrated in Figure 3. The reason is that for a relatively small *K* and *T*, the randomness in the one-bit measurement has not yet been eliminated by the summation in $S_{n}$. Therefore, in order to improve the image quality, additional steps must be taken to improve the smoothness of the image.

**Figure 4:**
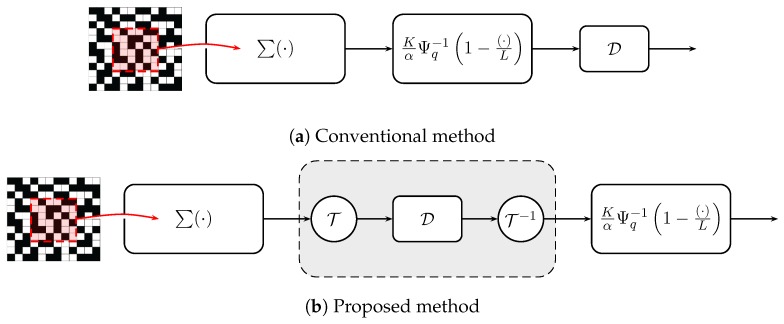
Two possible ways of improving image smoothness for QIS. (**a**) The conventional approach denoises the image after ${\hat{c}}_{n}$ is computed; (**b**) the proposed approach: apply the denoiser before the inverse incomplete Gamma function, together with a pair of Anscombe transforms T. The symbol D in this figure denotes a generic Gaussian noise image denoiser.

At first glance, this question seems easy because if one wants to mitigate the noise in $\hat{c}$, then directly applying an image denoising algorithm D to $\hat{c}$ would be sufficient, e.g., Figure 4a. However, a short afterthought will suggest that such an approach is invalid for the following reason. For the majority of image denoising algorithms in the literature, the noise is assumed to be independently and identically distributed (i.i.d.) Gaussian. In other words, the variance of the noise should be spatially invariant. However, the resulting random variable in Figure 2 does not have this property.

Our proposed solution is to apply an image denoiser before the inverse incomplete Gamma function as shown in Figure 4b. Besides the order of denoising and the Gamma function, we also add a pair of nonlinear transforms T and $T^{-1}$ before and after the denoiser D. The reasons for these two changes are based on the following observations.

**Observation** **1.** Under Assumption 1, the random variables $\left\{B_{Kn+k,t}\;|\;k=0,\ldots ,K-1,\;and\;t=0,\ldots ,\;T-1\right\}$ are i.i.d. Bernoulli of equal probability $P\left[B_{Kn+k,t}=1\right]=1-\Psi_{q}\left(\frac{\alpha c_{n}}{K}\right)$ for k=0,…,K−1 and t=0,…,T−1.

The proof of Observation 1 follows immediately from Assumption 1 that if $G=\left(1/K\right)I_{N\times N}⊗1_{K\times 1}$, we can divide the *M* jots into *N* groups each having K×T entries. Within the group, the one-bit measurements are all generated from the same pixel $c_{n}$.

The consequence of Observation 1 is that for a sequence of i.i.d. Bernoulli random variables, the sum is a Binomial random variable. This is described in Observation 2.

**Observation** **2.** *If $\left\{B_{Kn+k,t}\right\}$ are i.i.d. Bernoulli random variables with probability $P\left[B_{Kn+k,t}=1\right]=1-\Psi_{q}\left(\frac{\alpha c_{n}}{K}\right)$ for k=0,…,K−1 and t=0,…,T−1, then the sum $S_{n}$ defined in Equation* (12) *is a Binomial random variable with mean and variance:*

$$E\left[S_{n}\right]=L\left(1-\Psi_{q}\left(\frac{\alpha c_{n}}{K}\right)\right),\;\;\mathrm{Var}\left[S_{n}\right]=L\Psi_{q}\left(\frac{\alpha c_{n}}{K}\right)\left(1-\Psi_{q}\left(\frac{\alpha c_{n}}{K}\right)\right).$$

Observation 2 is a classic result in probability. The mean of the Bernoulli random variables is specified by the incomplete Gamma function $\Psi_{q}\left(\frac{\alpha c_{n}}{K}\right)$, which approaches one as *K* increases. Thus, for fixed *T*, the probability $1-\Psi_{q}\left(\frac{\alpha c_{n}}{K}\right)\to 0$ as K→∞. When this happens, the binomial random variable $S_{n}$ can be approximated by a Poisson random variable with mean $L\left(1-\Psi_{q}\left(\frac{\alpha c_{n}}{K}\right)\right)$ [38]. However, as *T* also grows, the binomial random variable $S_{n}$ can be further approximated by a Gaussian random variable due to the central limit theorem. Therefore, for a reasonably large *K* and *T*, the resulting random variable $S_{n}$ is approximately Gaussian.

The variance of this approximated Gaussian is, however, not constant. The variance changes across different locations *n* because $\mathrm{Var}[S_{n}]$ is a function of $c_{n}$. Therefore, if we want to apply a conventional image denoiser (which assumes i.i.d. Gaussian noise) to smooth $S_{n}$, we must first make sure that the noise variance is spatially invariant. The technique used to accomplish this goal is called the variance stabilizing transform [39]. In this paper, we use a specific variance stabilizing transform known as the Anscombe transform [32]. Anscombe transform is best known in the image processing literature for Poisson denoising, where one transforms observed Poisson data to approximately Gaussian with equal variance [24]. For binomial random variables $S_{n}$, the Anscombe transform and its property are given in Theorem 1.

**Theorem** **1** (Anscombe transform for binomial random variables)**.** *Let $S_{n}$ be a binomial random variable with parameters $\left(L,p_{n}\right)$, where $p_{n}=1-\Psi_{q}\left(\frac{\alpha c_{n}}{K}\right)$ and L=KT. Define the Anscombe transform of $S_{n}$ as a function T:{0,…,L}→R, such that*

(16) $$Z_{n}=T\left(S_{n}\right)\overset{\mathrm{def}}{=}\sqrt{L+\frac{1}{2}}\sin^{-1}\left(\sqrt{\frac{S_{n}+\frac{3}{8}}{L+\frac{3}{4}}}\right).$$

Then, the variance of $Z_{n}$ is $\mathrm{Var}\left[Z_{n}\right]=\frac{1}{4}+O\left(L^{-2}\right)$ for all n.

**Proof.**  The proof of Theorem 1 is given in the Appendix A. It is a simplified version of a technical report by Brown et al. [40]. The original paper by Anscombe [32] also contains a sketch of the proof. However, the sketch is rather brief, and we believe that a complete derivation would make this paper self-contained. ☐

The implication of Theorem 1 is that regardless of the location *n*, the transformed random variable $Z_{n}$ has a constant variance $\frac{1}{4}$ when *L* is large. Therefore, the noise variance is now location independent, and hence, a standard i.i.d. Gaussian denoiser can be used.

**Figure 5:**
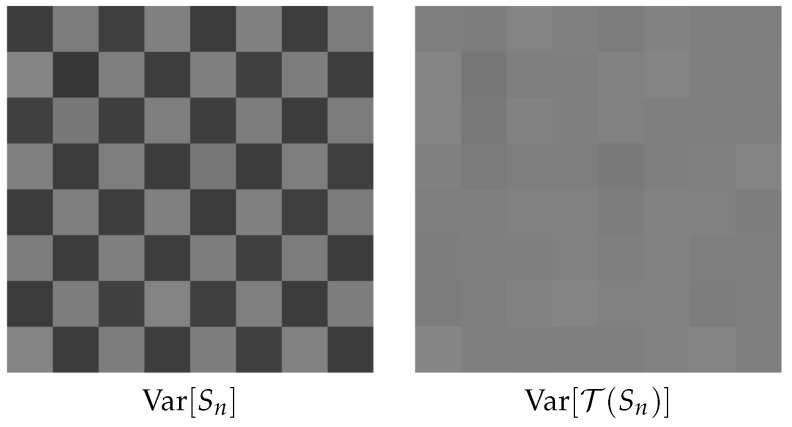
Illustration of Anscombe transform. Both sub-figures contain N=64 (8×8) pixels $c_{0},\ldots ,c_{N-1}$. For each pixel, we generate 100 binary Poisson measurements and sum to obtain binomial random variables $S_{0},\ldots ,S_{N-1}$. We then calculate the variance of each $S_{n}$. Note the constant variance after the Anscombe transform.

**Example** **2.** To provide readers a demonstration of the effectiveness of Theorem 1, we consider a checkerboard image of N=64 pixels with intensity levels $c_{0},\ldots ,c_{N-1}$. The n-th pixel $c_{n}$ generates K=100 binary quantized Poisson measurements $\left\{B_{Kn},\ldots ,B_{Kn+(K-1)}\right\}$ using α=100, q=1, T=1 (so L=100). From each of these K measurements, we sum to obtain a binomial random variable $S_{n}=\sum_{k=0}^{K-1}B_{Kn+k}$. We then compute the variance of $\mathrm{Var}[S_{n}]$ and $\mathrm{Var}[T\left(S_{n}\right)]$ using $10^{4}$ independent Monte Carlo trials. The results are shown in Figure 5, where we observe that $\mathrm{Var}[S_{n}]$ varies with the location n, and $\mathrm{Var}[T\left(S_{n}\right)]$ is nearly constant for all n.

**Remark** **1.** *The inverse Anscombe transform is*

(17) $$S_{n}=T^{-1}\left(Z_{n}\right)=\left(L+\frac{3}{4}\right)\sin^{2}\left(\frac{Z_{n}}{\sqrt{L+\frac{1}{2}}}\right)-\frac{3}{8},$$

*which we call the algebraic inverse. Another possible inverse of the Anscombe transform is the asymptotic unbiased inverse [32], defined as*

(18) $$S_{n}=T_{\mathrm{unbias}}^{-1}\left(Z_{n}\right)={\left(1+\frac{1}{2L}\right)}^{-1}\left[\left(L+\frac{3}{4}\right)\sin^{2}\left(\frac{Z_{n}}{\sqrt{L+\frac{1}{2}}}\right)-\frac{1}{8}\right].$$

*The performance of the unbiased inverse is typically better for low noise (large L), whereas the algebraic inverse is better for high noise (small L). This is consistent with the Poisson denoising literature. (e.g., [24])*.

**Example** **3.** Table 1 shows the peak signal to noise ratio (PSNR) values of the reconstructed images using the algebraic inverse and the asymptotic unbiased inverse. In this experiment, we consider 10 standard images commonly used in the image processing literature: Baboon, Barbara, Boat, Bridge, Couple, Hill, House, Lena, Man and Peppers. The sizes of the images are either 256×256 or 512×512. For each image, we set T=1, q=1 and α=K and vary K={1,4,9,16,25,36,49,64}. The results in Table 1 indicate that $T_{\mathrm{unbias}}^{-1}$ is consistently better than $T^{-1}$ for K>1, although the difference diminishes as K grows.

### 3.3. Component 3: Image Denoiser

An important feature of the proposed algorithm is that it can take any off-the-shelf image denoiser for the operator D. Here, by image denoiser, we meant an image denoising algorithm designed to remove i.i.d. Gaussian noise from an observed image.

Image denoising is an important research topic by its own. In the following, we provide a few popular image denoising algorithms.

- Total variation denoising [34]: Total variation denoising was originally proposed by Rudin, Osher and Fatemi [34], although other researchers had proposed similar methods around the same time [41]. Total variation denoising formulates the denoising problem as an optimization problem with a total variation regularization. Total variation denoising can be performed very efficiently using the alternating direction method of multipliers (ADMM), e.g., [42,43,44].
- Bilateral filter [45]: The bilateral filter is a nonlinear filter that denoises the image using a weighted average operator. The weights in a bilateral filter are the Euclidean distance between the intensity values of two pixels, plus the spatial distance between the two pixels. A Gaussian kernel is typically employed for these distances to ensure proper decaying of the weights. Bilateral filters are extremely popular in computer graphics for applications, such as detail enhancement. Various fast implementations of bilateral filters are available, e.g., [46,47].
- Non-local Means [48]: non-local means (NLM) was proposed by Buades et al. [48] and, also, an independent work of Awante and Whitaker [49]. Non-local means (NLM) is an extension of the bilateral filter where the Euclidean distance is computed from a small patch instead of a pixel. Experimentally, it has been widely agreed that such patch-based approaches are very effective for image denoising. Fast NLM implementations are now available [50,51,52].
- BM3D [53]: 3D block matching (BM3D) follows the same idea of non-local means by considering patches. However, instead of computing the weighted average, BM3D groups similar patches to form a 3D stack. By applying a 3D Fourier transform (or any other frequency domain transforms, e.g., discrete cosine transform), the commonality of the patches will demonstrate a group sparse behavior in the transformed domain. Thus, by applying a threshold in the transformed domain, one can remove the noise very effectively. BM3D is broadly regarded as a benchmark of today’s image denoising algorithm.

The factors to consider in choosing an image denoiser are typically the complexity and quality. Low complexity algorithms, such as bilateral filter, are fast, but the denoising ability is limited. High end algorithms, such as BM3D and non-local means, produce very good images, but require much computation. The trade-off between complexity and performance is a choice of the user.

**Figure 6:**
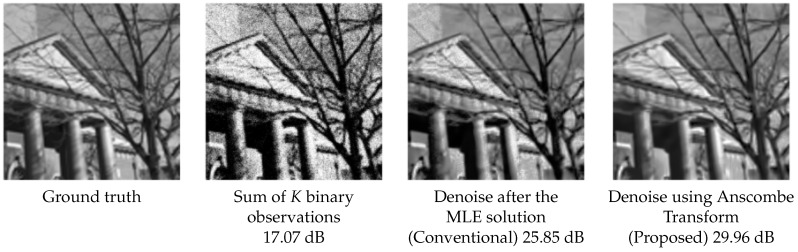
Comparison between image denoising after the MLE solution and using the proposed Anscombe transform. The denoiser we use in this experiment is 3D block matching (BM3D) [53]. The binary observations are generated using the configurations α=160, q=5, K=16, T=1. The values shown are the peak signal to noise ratio (PSNR).

Readers at this point may perhaps ask a question: What will happen if we apply a denoiser after the MLE solution, like the one shown in the block diagram in Figure 4a? As we have explained in the Anscombe transform section, this will lead to a suboptimal result because the noise is not i.i.d. Gaussian. To illustrate the difference in terms of performance, we show in Figure 6 a comparison between applying image denoising using the two block diagrams shown in Figure 4. The denoiser we use in this experiment is BM3D. The metric we use to evaluate the performance is the peak signal to noise ratio (PSNR), which will be defined formally in Section 4. In short, a large PSNR value is equivalent to a low mean squared error comparing the estimated image and the ground truth image. The results are shown in Figure 6. Although denoising after the MLE (the conventional idea) generates some reasonable images, the PSNR values are indeed significantly lower than the proposed Anscombe approach. This is not surprising, because the denoiser tends to oversmooth the dark regions and undersmooth the bright regions due to the signal-dependent noise levels.

### 3.4. Related Work in the Literature

The proposed algorithm belongs to a family of methods we call the transform-denoise methods. The idea of transform-denoise is similar to what we do here: transform the random variable using a variance stabilizing transform, then denoise using an off-the-shelf image denoiser. Among the existing transform-denoise methods, perhaps the most notable work is the one by Makitalo and Foi [24], where they considered the optimal inverse of the Anscombe transform for the case of Poisson–Gaussian random variables. A more recent work by the same research group [27] showed that it is possible to boost the denoising performance by applying the transform-denoise iteratively. We should also mention the work by Foi [54], which considered the modeling and transformation for clipped noisy images. The problem setting of that work is for conventional sensors. However, the underlying principle using the transform-denoise approach is similar to that of QIS.

The approximate MLE solution in Section 3.1 is based on the piecewise constant assumption (Assumption 1). Under this assumption, summing of the Bernoulli random variables can be thought of as performing a “binning” of the pixels. Binning is a common technique in restoring images from Poisson noise, especially when the signal-to-noise ratio is low [23,25,26]. Binning can also be applied together with transform-denoise, e.g., in [27], to achieve improved results. For QIS, the result of binning is different from that of the Poisson noise, for the sum of QIS bits leads to a binomial random variables, whereas the sum of Poisson noise leads to a Poisson random variable.

## 4. Experimental Results

In this section, we provide further experimental results to evaluate the proposed algorithm.

### 4.1. Synthetic Data

In this experiment, we consider 100 natural images downloaded from the Berkeley Segmentation database [55]. The input resolution of these images is 481×321 (or 321×481), and all images are converted to a gray-scale image with values in the range [0,1]. To generate the synthetic data, for each image, we consider an oversampling factor of four along the horizontal and the vertical directions (so K=16). The sensor gain is set as α=16, and the threshold level is q=1 to simulate a single photon sensor that triggers at one photon. We generate one-bit observations using a quantized Poisson statistics and use the proposed algorithm to reconstruct. As a comparison, we also test the MLE solution, i.e., a summation followed by the inverse incomplete Gamma transform (see Theorem 1), and an ADMM algorithm using a total variation regularization [29,31]. For the proposed algorithm, we use BM3D as the image denoiser.

**Figure 7:**
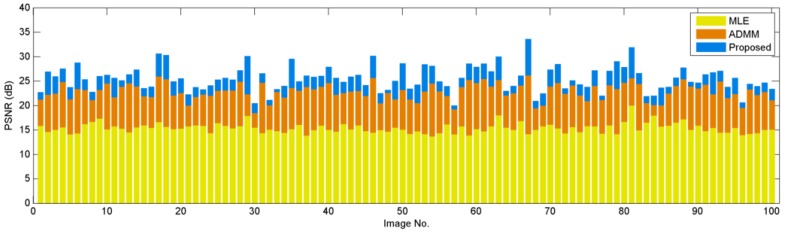
PSNR comparison of various image reconstruction algorithms on the Berkeley Segmentation database [55]. In this experiment, we fix q=1, α=16, and K=16. The proposed algorithm uses BM3D [53] as the image denoiser.

We report two results in this experiment. First, we consider the peak signal to noise ratio (PSNR) as an evaluation metric. The PSNR of an estimated image $\hat{c}$ compared to the ground truth image $c^{*}$ is defined as

(19) $$\mathrm{PSNR}=-20\log_{10}\left(\frac{∥\hat{c}-c^{*}{∥}^{2}}{N}\right),$$

where *N* is the number of pixels in c. Typically, higher PSNR values imply better image reconstruction quality. The PSNR values of these 100 images are shown in Figure 7. In this figure, we observe that the proposed algorithm is better than the MLE solution and the ADMM solution by 10.20 dB and 2.75 dB, respectively, which are very substantial amounts from a reconstruction perspective.

**Figure 8:**
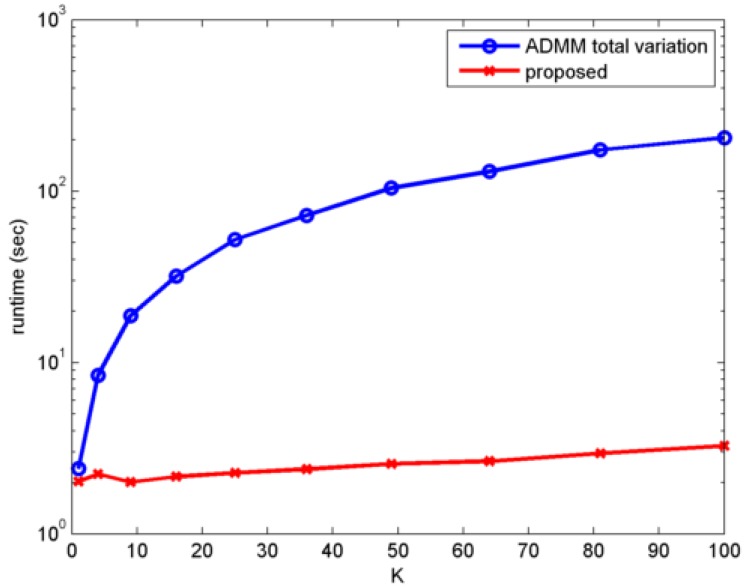
Runtime comparison of the proposed algorithm and the alternating direction method of multipliers (ADMM) algorithm [31].

Apart form the PSNR values, we also report the runtimes of the algorithms. The runtimes of the algorithms are recorded by running the methods on the same machine and the same platform, which is an Intel i7-6700 3.4-GHz desktop with Windows 7/MATLAB 2014. As shown in Figure 8, the runtime of the proposed algorithm is approximately two orders of magnitude (100×) faster than the ADMM algorithm.

**Figure 9:**
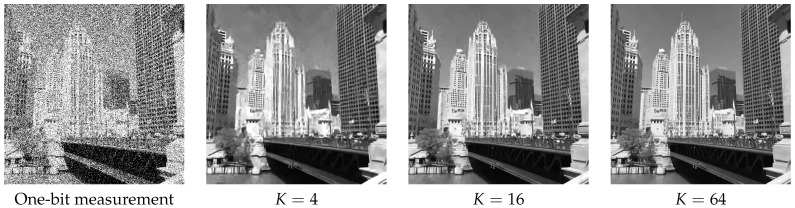
Influence of the oversampling factor *K* on the image reconstruction quality. In this experiment, we set α=K, q=1. T=1.

As for the influence of the oversampling factor *K* on the reconstruction quality, we show in Figure 9 the reconstructed results using K=4,16,64 and the binary one-bit measurements. While it is clear from the figure that the reconstructed image improves as *K* increases, we observe that most of the visual content has been recovered even at K=4.

### 4.2. Real Data

In this experiment, we consider two single-photon avalanche diode (SPAD) cameras for capturing high speed videos. The first camera is a CMOS SPAD-based image sensor developed by Dutton et al. [14,15,16]. This camera has a resolution of 320×240, with a frame rate of 10k frames per second. The second camera is the SwissSPAD camera developed by Burri et al. [17,18]. This camera has a resolution of 512×128, with frame rate of 156k frames per second. Both cameras capture one-bit measurements from a scene containing a stationary background with a rapidly moving foreground. Our experimental goal is to test if the proposed algorithm can resolve the spatial content with minimal trade-off in the temporal resolution.

There are several points of this experiment on which we should comment. First, since the spatial resolution of these two cameras is relatively small (as compared to the synthetic case), we do not assume any spatial oversampling, i.e., K=1. Instead, we use *T* temporal frames to reconstruct one output image. To ensure smooth transitions across adjacent frames, we use a temporally-sliding window as we progress to the next output image. Second, since these real videos do not have a ground truth, we can only compare the quality of the resulting images visually.

**Figure 10:**
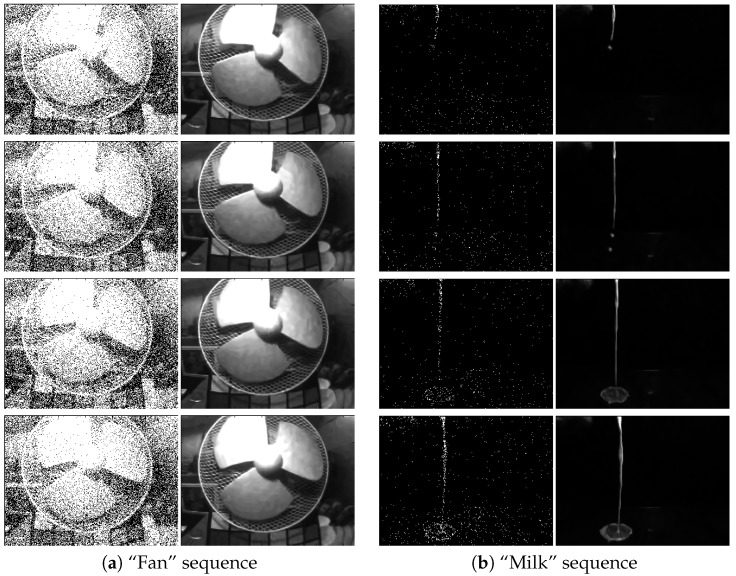
Image reconstruction of two real video sequences captured using a 320×240 single-photon avalanche diode (SPAD) camera running at 10k frames per second [14,15,16]. In this experiment, we use T=16 frames to construct one output frame. In both columns, the left are the raw one-bit measurements, and the right are the recovered images using the proposed algorithm.

We first look at the results of the SPAD camera by Dutton et al. [14,15,16]. Figure 10 shows several snapshots of the “fan” sequence and the “milk” sequence. To generate this result, we run the proposed algorithm using T=16 frames in a sliding window mode. That is, we use Frames 1 to 16 to recover Frame 1 and Frames 2 to 17 to recover Frame 2, etc. The quantization level *q* is set as q=1, and the sensor gain *α* is adjusted to produce the best visual quality. For the “fan” sequence, we set α=4, and for the “milk” sequence, we set α=0.75. As we can see from the figures, the proposed algorithm recovers most of the content from the scene, even revealing the textures of the milk in the scene.

**Figure 11:**
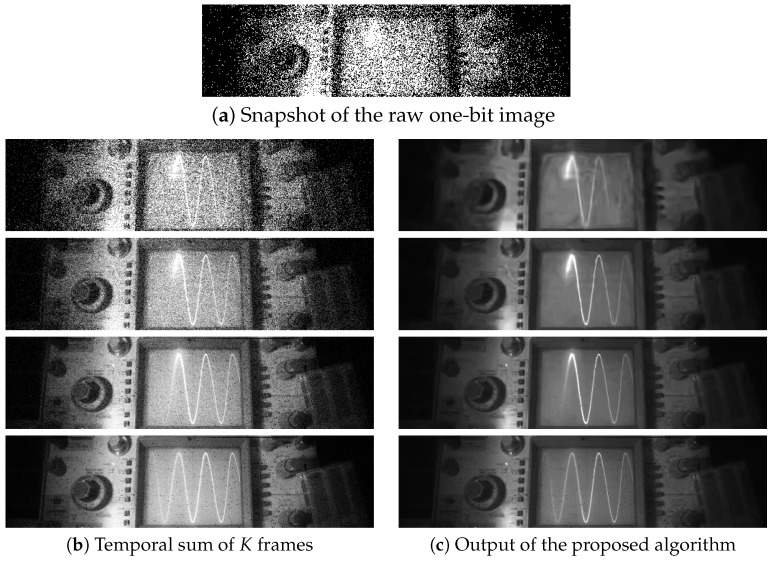
Image reconstruction of real video sequences captured using the 512×128 SwissSPAD camera running at 156k frames per second [17,18]. (**a**) is a snapshot of the raw one-bit image. (**b**) shows the result of summing *T* = 4, 16, 64, 256 temporal frames with K=1. (**c**) shows the corresponding results using the proposed algorithm.

As for the SwissSPAD camera, we consider a video sequence “oscilloscope” captured at a frame rate of 156k frames per second. The goal is to track the sinusoid shown on the oscilloscope’s screen. We consider four values of *T* = 4, 16, 64 and 256 for the number of frames. The results are shown in Figure 11. As one may expect, when T=256, the image quality improves because we are effectively summing 256 frames to reconstruct one output frame. However, since we are summing the 256 frames, the temporal resolution is severely distorted. In particular, the trace of the sinusoid signal disappears because of the strong averaging effect. When we reduce the number of frames to T=16, we observe that the trace of the signal can be observed clearly. The same image obtained by the MLE (i.e., simple summation) is still highly noisy.

## 5. Conclusions

We present a new image reconstruction algorithm to recover images from one-bit quantized Poisson measurements. Different from existing algorithms that are mostly iterative, the new algorithm is non-iterative. The algorithm consists of three key components: (1) an approximation to the standard maximum likelihood estimation formulation that allows us to decouple the dependency of pixels; (2) a nonlinear transform known as the Anscombe transform that converts a sum of one-bit quantized Poisson random variables to a Gaussian random variable with equal variance; (3) an off-the-shelf image denoising algorithm that performs the smoothing. Experimental results confirm the performance of the proposed algorithm. The algorithm demonstrates two orders of magnitude improvement in speed compared to existing iterative methods and shows several dBs of improvement in terms of the peak signal to noise ratio (PSNR) as a metric of image quality.

## Figures and Tables

**Table 1:** PSNR values using algebraic inverse $T^{-1}$ and asymptotic unbiased inverse $T_{\mathrm{unbias}}^{-1}$. The results are averaged over 10 standard images. In this experiment, we set T=1, q=1 and α=K.

K	1	4	9	16	25	36	49	64
$T^{-1}$	20.51	23.08	25.00	26.47	27.49	28.40	29.09	29.71
$T_{\mathrm{unbias}}^{-1}$	19.43	23.64	25.30	26.62	27.57	28.45	29.12	29.73

## Abbreviations

The following abbreviations are used in this manuscript:

CCD	Charge coupled device
CMOS	Complementary metal-oxide-semiconductor
QIS	Quanta image sensors
MLE	Maximum likelihood estimation
ADMM	Alternating direction method of multipliers
SPAD	Single-photon avalanche diode
PSNR	Peak signal to noise ratio
BM3D	3D block matching
i.i.d.	Independently and identically distributed

## Appendix A

### Appendix A.1. Proof of Proposition 1

**Proof.** For notational simplicity, we drop the subscript *n*. Then, the optimization problem is

$$\hat{c}=\underset{c}{\mathrm{argmax}}\;\;S\log\left(1-\Psi_{q}\left(\frac{\alpha c}{K}\right)\right)+\left(L-S\right)\log\Psi_{q}\left(\frac{\alpha c}{K}\right).$$

Taking the first order derivative with respect to *c* and setting to zero yields (with the help of Property 1):

$$\begin{array}{c} \left(\frac{S}{1-\Psi_{q}\left(\frac{\alpha c}{K}\right)}\right)\left(\frac{\alpha }{K}\frac{e^{-\frac{\alpha c}{K}}{\left(\frac{\alpha c}{k}\right)}^{q-1}}{\Gamma (q)}\right)+\left(\frac{L-S}{\Psi_{q}\left(\frac{\alpha c}{K}\right)}\right)\left(-\frac{\alpha }{K}\frac{e^{-\frac{\alpha c}{K}}{\left(\frac{\alpha c}{K}\right)}^{q-1}}{\Gamma (q)}\right)=0. \end{array}$$

Rearranging the terms leads to the desired result that

$$\Psi_{q}\left(\frac{\alpha c}{K}\right)=1-\frac{S}{L}.$$

☐

### Appendix A.2. Proof of Theorem 1

**Proof.** For notational simplicity, we drop the subscript *n*. Our goal is to show that if *X*∼Binomial(L,p), then the transformed variable:

(A1) $$T\left(X\right)=\sqrt{L+\frac{1}{2}}\sin^{-1}\left(\sqrt{\frac{X+\frac{3}{8}}{L+\frac{3}{4}}}\right)$$

has a variance $\mathrm{Var}\left[T\left(X\right)\right]=\frac{1}{4}+O\left(L^{-2}\right)$. To this end, we first consider the function Q, such that

$$Q\left(X\right)=T\left(X\right)-\sqrt{L+\frac{1}{2}}\sin^{-1}\sqrt{p}.$$

Since Var[Q(X)+c]=Var[Q(X)] for any *c*, by letting $c=-\sqrt{L+\frac{1}{2}}\sin^{-1}\sqrt{p}$, we observe that showing Proposition 1 is equivalent to showing $\mathrm{Var}\left[Q\left(X\right)\right]=\frac{1}{4}+O\left(L^{-2}\right)$. To show the desired result, we note that for any *α* and *β*, the arcsin function has the property that

$$\sin^{-1}\alpha -\sin^{-1}\beta =\sin^{-1}\left(\alpha \sqrt{1-\beta^{2}}-\beta \sqrt{1-\alpha^{2}}\right).$$

Define $F\overset{\mathrm{def}}{=}\frac{X+\frac{3}{8}}{L+\frac{3}{4}}$, and substitute $\alpha =\sqrt{F}$, $\beta =\sqrt{p}$; it follows that

(A2) $$Q\left(X\right)=\sqrt{L+\frac{1}{2}}\sin^{-1}\left(\sqrt{(1-p)F}-\sqrt{p(1-F)}\right).$$

There are two terms in this equation. The first term $\sqrt{L+\frac{1}{2}}$ can be expanded (using Taylor expansion) to its first second order as

$$\begin{array}{cc} \sqrt{L+\frac{1}{2}} & =\sqrt{L}{\left(1+\frac{1}{2L}\right)}^{\frac{1}{2}}=\sqrt{L}\left(1+\frac{1}{4L}+O\left(L^{-2}\right)\right). \end{array}$$

The arcsin function can be expanded to its second order as

$$\begin{array}{c} \sin^{-1}W=W+\frac{W^{3}}{6}+\frac{3W^{5}}{40}+\frac{5W^{7}}{112}+\ldots , \end{array}$$

for $W=\sqrt{(1-p)F}-\sqrt{p(1-F)}$. We next consider the standardized binomial random variable by defining

(A3) $$Y\overset{\mathrm{def}}{=}\frac{X-Lp}{\sqrt{Lp(1-p)}}.$$

Then, by Lemma A1, it follows that

$$\begin{array}{cc} W & =\sqrt{(1-p)F}-\sqrt{p(1-F)}\\ & =\frac{Y}{2\sqrt{L}}+\frac{\left(2p-1\right)\left(2Y^{2}-3\right)}{16L\sqrt{p(1-p)}}+\frac{-16Y^{3}p^{2}+16Y^{3}p-6Y^{3}+9Y}{96L^{\frac{3}{2}}p\left(1-p\right)}+O\left(L^{-2}\right). \end{array}$$

Therefore,

$$\begin{array}{cc} Q(X) & =\sqrt{L}\left(1+\frac{1}{4L}+O\left(L^{-2}\right)\right)\left(W+\frac{W^{3}}{6}+O\left(W^{5}\right)\right)\\ & =a_{0}+a_{1}Y+a_{2}Y^{2}+a_{3}Y^{3}+O\left(Y^{5}\right), \end{array}$$

where

$$\begin{array}{c} \begin{array}{cc} a_{0}=-\frac{3(2p-1)}{16\sqrt{Lp(1-p)}}, & a_{1}=\frac{1}{2}+\frac{1}{8L}-\frac{3}{32Lp(1-p)}\\ a_{2}=\frac{2p-1}{8\sqrt{Lp(1-p)}}, & a_{3}=\frac{16p^{2}-16p+6}{96Lp(1-p)}. \end{array} \end{array}$$

Since the first four moments of *Y* are

$$\begin{array}{c} E\left[Y\right]=0,\;\;E\left[Y^{2}\right]=1,\;\;E\left[Y^{3}\right]=-\frac{2p-1}{\sqrt{Lp(1-p)}},\;\;E\left[Y^{4}\right]=3+\frac{1-6p(1-p)}{Lp(1-p)}, \end{array}$$

we conclude that

$$\begin{array}{cc} \mathrm{Var}[Q(X)] & =a_{1}^{2}\mathrm{Var}\left[Y\right]+a_{2}^{2}\mathrm{Var}\left[Y^{2}\right]+2a_{1}a_{2}\mathrm{Var}\left[Y^{3}\right]+2a_{1}a_{3}\mathrm{Var}\left(Y^{4}\right)\\ & =a_{1}^{2}-a_{2}^{2}+2a_{1}a_{2}E\left[Y^{3}\right]+\left(2a_{1}a_{3}+a_{2}^{2}\right)E\left[Y^{4}\right]=\frac{1}{4}+O\left(L^{-2}\right). \end{array}$$

 ☐

**Lemma** **A1.** *Let $F=\frac{X+\frac{3}{8}}{L+\frac{3}{4}}$ and $Y=\frac{X-Lp}{\sqrt{Lp(1-p)}}$. It holds that*

(A4) $$\begin{array}{cc} \sqrt{(1-p)F} & =\sqrt{p(1-p)}+\frac{(1-p)Y}{2\sqrt{L}}-\frac{\sqrt{1-p}\left(6p+2\left(1-p\right)Y^{2}-3\right)}{16L\sqrt{p}}\\ & \;\;-\frac{\left(1-p\right)Y(6p-2\left(1-p\right)Y^{2}+3)}{32pL^{\frac{3}{2}}}+O\left(L^{-2}\right). \end{array}$$

(A5) $$\begin{array}{cc} \sqrt{p(1-F)} & =\sqrt{p(1-p)}-\frac{pY}{2\sqrt{L}}-\frac{\sqrt{p}\left(-6p+2pY^{2}+3\right)}{16L\sqrt{1-p}}\\ & \;\;-\frac{pY(6p+2pY^{2}-9)}{32\left(1-p\right)L^{\frac{3}{2}}}+O\left(L^{-2}\right). \end{array}$$

**Proof.** Note that $Y=\frac{X-Lp}{\sqrt{Lp(1-p)}}$ is equivalent to $X=Y\sqrt{Lp(1-p)}+Lp$. Thus, *F* can be expressed in terms of *Y* as

$$F=\frac{\left(Y\sqrt{Lp(1-p)}+Lp\right)+\frac{3}{8}}{L+\frac{3}{4}}=\left(Y\sqrt{\frac{p(1-p)}{L}+p+\frac{3}{8L}}\right){\left(1+\frac{3}{4L}\right)}^{-1}.$$

For large *L*, we have $\frac{3}{4L}\ll 1$. Thus, by expanding ${\left(1+\frac{3}{4L}\right)}^{-1}$, we have

$$\begin{array}{cc} F & =\left(Y\sqrt{\frac{p(1-p)}{L}+p+\frac{3}{8L}}\right)\left(1-\frac{3}{4L}+O\left(L^{-2}\right)\right)=p\left(1+E_{1}\right), \end{array}$$

where

$$E_{1}=\sqrt{\frac{1-p}{p}}\frac{Y}{\sqrt{L}}-\frac{\frac{3}{4}-\frac{3}{8p}}{L}-\sqrt{\frac{p}{1-p}}\frac{3Y}{4L^{\frac{3}{2}}}+O\left(L^{-2}\right).$$

By expanding $\sqrt{1+E_{1}}$, we arrive at

$$\begin{array}{cc} \sqrt{F} & =\sqrt{p}\sqrt{1+E_{1}}=\sqrt{p}\left(1+\frac{E_{1}}{2}-\frac{E_{1}^{2}}{8}+\frac{E_{1}^{3}}{16}+O\left(E_{1}^{4}\right)\right). \end{array}$$

Multiplying both sides by $\sqrt{1-p}$ and substituting for $E_{1}$ yields

$$\begin{array}{cc} \sqrt{(1-p)F} & =\sqrt{p(1-p)}+\frac{(1-p)Y}{2\sqrt{L}}-\frac{\sqrt{1-p}\left(6p+2\left(1-p\right)Y^{2}-3\right)}{16L\sqrt{p}}\\ & \;\;-\frac{\left(1-p\right)Y(6p-2\left(1-p\right)Y^{2}+3)}{32pL^{\frac{3}{2}}}+O\left(L^{-2}\right). \end{array}$$

The proof of the second equality can be done by expressing 1−F in terms of *Y* as

$$\begin{array}{cc} 1-F & =\left(-Y\sqrt{\frac{p(1-p)}{L}}+\left(1-p\right)+\frac{3}{8L}\right){\left(1+\frac{3}{4L}\right)}^{-1}.\\ & =\left(-Y\sqrt{\frac{p(1-p)}{L}}+\left(1-p\right)+\frac{3}{8L}\right)\left(1-\frac{3}{4L}+O\left(L^{-2}\right)\right)=\left(1-p\right)\left(1+E_{2}\right), \end{array}$$

where

$$E_{2}=-\sqrt{\frac{p}{1-p}}\frac{Y}{\sqrt{L}}-\frac{\frac{3}{4}-\frac{3}{8(1-p)}}{L}+\sqrt{\frac{1-p}{p}}\frac{3Y}{4L^{\frac{3}{2}}}+O\left(L^{-2}\right).$$

By expanding $\sqrt{1+E_{2}}$, we arrive at

$$\begin{array}{cc} \sqrt{1-F} & =\sqrt{1-p}\sqrt{1+E_{2}}=\sqrt{1-p}\left(1+\frac{E_{2}}{2}-\frac{E_{2}^{2}}{8}+\frac{E_{2}^{3}}{16}+O\left(E_{2}^{4}\right)\right). \end{array}$$

Multiplying both sides by $\sqrt{p}$ and substituting for $E_{2}$ yields

$$\begin{array}{cc} \sqrt{p(1-F)} & =\sqrt{p(1-p)}-\frac{pY}{2\sqrt{L}}-\frac{\sqrt{p}\left(-6p+2pY^{2}+3\right)}{16L\sqrt{1-p}}\\ & \;\;-\frac{pY(6p+2pY^{2}-9)}{32\left(1-p\right)L^{\frac{3}{2}}}+O\left(L^{-2}\right). \end{array}$$

☐
